# Effects of Nano Emulsified Vegetable Oil and Betaine on Growth Traits and Meat Characteristics of Broiler Chickens Reared under Cyclic Heat Stress

**DOI:** 10.3390/ani11071911

**Published:** 2021-06-27

**Authors:** Alaeldein M. Abudabos, Gamaleldin M. Suliman, Abdullah N. Al-Owaimer, Ali R. Al Sulaiman, Abdulrahman S. Alharthi

**Affiliations:** 1 Department of Animal Production, College of Food and Agriculture Sciences, King Saud University, P.O. Box 2460, Riyadh 11451, Saudi Arabia; gsuliman@ksu.edu.sa (G.M.S.); Aowaimer@ksu.edu.sa (A.N.A.-O.); 2 National Center for Environmental Technology, Life Science and Environment Research Institute, King Abdulaziz City for Science and Technology, P.O. Box 6086, Riyadh 11442, Saudi Arabia; arsuliman@kacst.edu.sa

**Keywords:** broiler, cyclic heat stress, thermoneutral, meat characteristic, performance, betaine, nano emulsified vegetable oil

## Abstract

**Simple Summary:**

Thermal stress is a critical issue for poultry during the summertime. It is imperative to overcome this problem by controlling the environment inside poultry houses or by water additives. This experiment evaluated the effects of two supplements for broilers in water. Nano-emulsified vegetable oil or betaine was used in two groups of broilers. One group was kept under a thermo-neutral temperature (24 °C) while the other was kept under a high temperature (35 °C). The high temperature lasted from 21 to 35 days old. The high-temperature group consumed less feed and suffered significantly from lower daily gain. Moreover, the thermo-neutral group had good meat attributes when compared to the high-temperature group. The two supplements improved the ability of broilers to use feed more efficiently and convert it to body gain compared to the un-supplemented birds. In addition, betaine enhanced carcass characteristics such as breast temperature and pH compared to the control. In summary, the heat challenge caused significant disadvantageous impacts on growth efficiency and meat qualities, while the two supplements used herein improved the performance. Therefore, it is recommended to use these supplements in the case of high temperatures inside broiler houses.

**Abstract:**

The effects of nano-emulsified vegetable oil (NEVO) and betaine (BET) supplements on growth performance and meat qualities of broilers reared under cyclic heat stress (HS) were investigated. Two hundred and eighty-eight mixed-sex broilers at 21 d were randomly distributed to a 2 × 3 factorial arrangement of treatments formed by two environmental temperatures (thermoneutral (TN; 24 ± 1 °C) and cyclic high-temperature (HT; 35 ± 1 °C)) and three dietary treatments (control (CON), NEVO, and BET). The cumulative performance (21–35 d) revealed a reduction in average daily gain (ADG) (*p* < 0.05) in the CON compared to NEVO. NEVO and BET groups had a better feed conversion ratio (FCR) and performance efficiency factor (PEF) compared with the CON (*p* < 0.001, *p* < 0.01, respectively). The environmental temperature affected daily feed intake (DFI), ADG, FCR, and PEF. The addition of BET improved breast fillets yield, temperature, pH_15min,_ and pH_24hr_ (*p* < 0.05) in comparison with the CON. Moreover, the TN group had lower fillet temperature and higher pH_15min_ compared to the HT. Moreover, HT increased shear force (SF), hardness, springiness, cohesiveness, and chewiness of the fillets compared to TN. In conclusion, dietary supplementation with BET and NEVO could effectively improve performance parameters and meat characteristics under HS conditions.

## 1. Introduction

Genetic improvements of modern broiler strains have selected a rapid muscle growth rate at the cost of the functional efficacy of cardiovascular and thermoregulatory systems [1]. As a result, nowadays, broilers have less ability to control their body heat during high environmental temperature (HT) than slow-growing broilers [2]. Modern strains of broilers with higher internal heat production are more vulnerable to HT with advanced age due to high metabolic activity and the absence of sweat glands [3]. Furthermore, commercial broiler production is very concentrated with elevated stocking density, an amalgamation of these circumstances bringing about an increment in heat accumulation within broiler houses [4]. In tropical and subtropical areas, HT is one of the most significant factors prompting stress in broiler production [5]. Plentiful studies on broilers have demonstrated that HS would induce numerous physiological and metabolic instabilities, such as endocrine disorders [6,7], electrolyte imbalance [8,9], immune suppression [10,11], and oxidative damage in various tissues [12,13]. In an effort to remove internal heat, broilers increase respiratory rate under HS conditions, and as a result, acid-base imbalance or alkalosis occurs [14]. Prolonged HS affects feed digestion and absorption as a consequence of blood flow reduction to the digestive system, and consequential injuries in intestinal-epithelial tissues could occur [15]. Moreover, prolonged HS may also upset the intestinal barrier, increasing the possibility of entering bacterial pathogens and endotoxins into the bloodstream, which thereafter can bring on exaggerated inflammations [16].

One strategy to solve the HS problem is improving environmental management; another strategy is nutritional approaches, including changing dietary energy levels and supplementing salts, antioxidant vitamins, and minerals [17]. Currently, nanoemulsions supplements in water have also been implemented in poultry to counteract the adverse effects of HS [18].

Betaine (BET) is incorporated commercially in poultry feeds and drinking water in the forms of hydrochloride and anhydrous [19]. Functionally, BET acts as an osmoprotectant for cellular water homeostasis and as a methyl group donor for the methionine-homocysteine cycle [20,21]. Furthermore, Alirezaei et al. [22] and Nasiroleslami et al. [23] found that BET progressed antioxidative defense in broilers who experienced stressful circumstances through the methionine’s cycles. Betaine, when examined as a feed or water additives, showed that it has several advantages to the broiler industry, involving enhanced intestinal morphology [24,25], improved antioxidant defenses, and decreased lipid peroxidation in the breast muscles [22,26], and improved carcass composition by changing lipid metabolism [27,28].

Another approach to overcome the problem of low feed intake during HS is to utilize nanoemulsions, which can simply be formulated and administered as an energy supplement, particularly during HS. Nanoemulsions are made up of tiny oil droplets spread in water; they are employed as transported systems for several hydrophobic materials such as nutrients, nutraceuticals, antioxidants, and antimicrobials [29]. Structurally, nanoemulsions can be categorized based on the relative sites of the dispersed and continuous stages [30]. It was reported that broilers supplemented with nano-emulsified vegetable oil (NEVO) had a considerable improvement in body weight and feed conversion ratio (FCR) [31]. Accordingly, the present research was carried out to examine the disadvantageous influence of cyclic HS on performance and meat characteristics in broilers and to evaluate whether feeding BET and NEVO could ameliorate the adverse conditions induced by HS.

## 2. Materials and Methods

### 2.1. Institutional Review Board Statement

The study was conducted according to the guidelines of the Declaration of Helsinki and approved by the Ethics Committee of King Saud University, Saudi Arabia (KSU-SE-20–22).

### 2.2. Birds, Diets, and Experimental Design

Chicks (Ross 308) were obtained commercially; they were vaccinated for Newcastle disease, Marek’s disease, and infectious bronchitis at the hatchery. All chicks received commercial starter and grower diets (0–20 d). Chicks were maintained under recommended environmental temperatures; the initial brooding temperature on day 0 was set at 33 ± 1 °C and was decreased weekly till 24 ± 1 °C on d 20. At 21 d old, a total of 288 straight run broiler chicks with comparable body weight (804 g ± 0.66) were selected and randomly allocated into 36-floor cages (100 cm length, 100 cm width) in two environmentally controlled rooms. Each cage with eight birds was considered as the experimental unit. A 23 h artificial light in both rooms was used throughout the experimental period. According to the strain’s nutritional recommendations, experimental diets were formulated for the finisher phase (21–35 d) (Table 1).

During the finisher period, a 2 × 3 factorial arrangement was applied. Six experimental groups (6 replicates per group) were formed by using two environmental temperatures (thermoneutral environment (TN) and cyclic high-temperature (HT) conditions) and three dietary treatments (control (CON), NEVO, and BET). The birds in one of the two experimental rooms were continuously kept in the TN environment (24 ± 1 °C, TN group), while birds in the second room were raised under cyclic HT conditions (35 ± 1 °C, HS group) from 9.00 am to 5.00 pm (8 h daily) followed by TN temperature for the rest of the period. The HS treatment lasted for 14 successive days until 35 d. The maximum and minimum ambient temperatures of the experimental rooms were constantly monitored using data loggers (HOBO Pro Series, Model H08-032-08, ONSET Co., Cape Cod, MA, USA). The three dietary treatments used were CON (no additive), NEVO at the rate of 5 mL/L drinking water, and BET at the rate of 2 g/L drinking water. The six treatment groups were as follows: group 1, TN + CON; group 2, HT + CON; group 3, TN + 5 mL/L drinking water NEVO; group 4, HT + 5 mL/L drinking water NEVO; group 5, TN + 2 g/L drinking water; and group 6, HT + 2 g/L drinking water. NEVO and BET were dissolved in 50 L buckets, and 12 L waterers were filled daily. NEVO consists of polysorbate-80, vitamin E, and soybean oil (ATCO Pharma, Egypt). BET (^TNI^betaine 96%, anhydrous) was obtained from Selko feed additives (Trouw Nutrition, Nutreco Company, Rotterdam, The Netherlands).

### 2.3. Performance, Carcass, and Meat Determinations

Daily feed intake (DFI) and average daily gain (ADG) were estimated by measuring feeders and birds on a pen basis at 7 d periods; the FCR and European performance efficiency factor (PEF) were then determined.

At 35 d, ten birds with body weights similar to the group’s average were randomly selected per treatment and weighed individually after 12 h fasting. The birds were then slaughtered by severing the jugular vein, the carcass was de-feathered and autopsied, the internal and lymphoid organs were separated and measured, dressing percentage and parts yield were also determined, and then the relative weight (g/100 g of BW) were calculated.

The left breast was utilized for meat quality as published by Al-Owaimer et al. [32]. Briefly, internal breast temperature was estimated at 15 min post-mortem with a transportable digital thermocouple. The pH was estimated in duplicate at 15 min and 24 h post-mortem by placing a pH probe 2.0 cm beneath the pectoral muscles.

Color grades were assessed at 15 min and 24 h post-mortem employing a CR-400 Chromameter (Konica Minolta, Tokyo, Japan). Values of L* (lightness), a* (redness), and b* (yellowness) were used to calculate color change (∆E*), color saturation (C*), hue angle (H°) and a*: b* proportion. The next equalizations were used for calculations: ∆E* = ((∆L*)^2^ + (∆a*)^2^ + (∆b*) ^2^)^0.5^, C* = (a*^2^ + b*^2^)^0.5^, and H° = tan−1 (b*/a*) [33,34].

The pectoralis major muscle was cooked on a countertop grille till the geometric center temperature, which was checked with a thermocouple thermometer, reached 70 °C [35]. Cooking loss (CL) was determined as a ratio between the sample’s initial and final weights. Water holding capacity (WHC) was evaluated via placing 2 g of the cranial part of the breast fillet betwixt two segments of filter paper and two segments of Plexiglass and placed beneath a 10 kg weighing for 5 min; WHC was then measured by calculating the difference between the original and eventual weights [36]. Myofibril fragmentation index (MFI) was measured following homogenizing 4 g of minced sample in 40 mL of a chilled MFI buffer by estimating the absorbance with 540 nm [37]. The cores of cooked muscles were removed aligned to the longitudinal direction of the myofibers, and shear force (SF) was afterward estimated by determining the maximal strength in kg perpendicularly to the fibers employing a Texture Analyzer furnished with a Warner–Bratzler attachment [38].

The cooked specimen was utilized to estimate the texture profile analysis (TPA) by employing a Texture Analyzer (Stable Micro Systems, Godalming, Surrey GU7 1YL, UK) provided with a compression-plate attachment. Springiness was estimated as the capability of a specimen to return to its primary configuration following eliminating the compressive force. Hardness was evaluated as the maximal force required to squeeze the specimen. Cohesiveness was assessed as the proportion of the gross power demanded for the initial and subsequent squeezing. The chewiness was calculated by the multiplication of (springiness × hardness × cohesiveness).

### 2.4. Statistical Analysis

The data from the finisher period were subjected to 2-way ANOVA as a 2 × 3 factorial arrangement of treatments, using the GLM procedure of SAS software, Release 9.1 for windows, (2003) to assess the main effects of diet, temperature, and any possible interactions. Significant values based on *p*-value ≤ 0.05 were segregated employing Tukey’s range test. The results obtained were presented as least squares means and their pooled standard error of mean (SEM).

## 3. Results

### 3.1. Growth Performance

Performance parameters are presented in Table 2. For the period (21–28 d), the results showed that the two-way interaction of TEMP and DIET was not significant for any parameter for this period (*p* > 0.05). Differences in DFI and ADG were not significant due to diet (*p* > 0.05). However, FCR and PEF were improved due to NEVO and BET compared to the CON (*p* < 0.001). Environmental temperature affected ADG and PEF (*p* < 0.05), birds subjected to HT had lower ADG and PEF compared to the TN group.

For the period (28–35 d), the results revealed no significant two-way interaction of TEMP and DIET (*p* > 0.05). The difference in DFI was insignificant due to diet (*p* > 0.05). The ADG, FCR, and PEF were improved due to NEVO and BET compared to the CON (*p* < 0.05, *p* < 0.001, *p* < 0.001, respectively). Environmental temperature affected ADG, FCR, and PEF (*p* < 0.01), and birds subjected to HT had lower ADG and PEF and higher FCR compared to the TN group.

For the cumulative finisher period (21–35 d), the results showed no significant two-way interaction between TEMP and DIET (*p* > 0.05). A lower ADG (*p* < 0.05) was obtained by CON compared to NEVO, but it was insignificant to BET. Significantly worse FCR and PEF were associated with the CON compared to NEVO and BET (*p* < 0.001, *p* < 0.01, respectively). DFI, ADG, FCR, and PEF (*p* < 0.05, *p* < 0.01, *p* < 0.05, *p* < 0.05, respectively) were affected by temperature; the birds subjected to HT had lower DFI, ADG, and PEF and higher FCR compared to TN group.

### 3.2. Carcass Parts and Internal Organs

The weights of the cut-up parts and internal organs (g/100 g of live weight) of broiler birds at 35 d are shown in Table 3. No significant two-way interaction of TEMP and DIET was detected (*p* > 0.05). BET supplementation enhanced breast muscle yield over the CON (*p* < 0.05). Moreover, HT decreased breast yield when compared to TN (*p* < 0.05). The liver percentage was affected by diet (*p* < 0.05); the BET supplementing group had a lower liver percentage compared to the CON or NEVO groups. Moreover, the intestine relative weight (IRW) percentage was lower for BET (*p* < 0.05) compared to CON. The bursa of *Fabricius* was lower for the HT group (*p* < 0.05) compared with the TN group. At the same time, broilers in BET and NEVO groups had higher (*p* < 0.01) relative weights of the bursa of *Fabricius* compared to the CON group.

### 3.3. Meat Quality

The results for breast meat quality traits are shown in Table 4. There were no significant interaction effects on meat quality parameters in breast muscle. At 15 min post-mortem, diet affected breast fillets temperature and pH (*p* < 0.05), and the CON had a higher breast fillets temperature compared to BET while NEVO had an intermediate temperature. In addition, breast fillets from the BET group had higher pH than the CON and NEVO groups. No treatment effect (*p* > 0.05) was observed in L*, a*, b*, C*, H°, and a*/b* ratio when measured at that time. On the other hand, environmental temperature affected breast fillets temperature (*p* < 0.001) and pH (*p* < 0.01); the TN group had lower fillets temperature and higher pH, compared to HT. At 24 h post-mortem, diet only affected breast fillets pH (*p* < 0.01); the CON had lower breast pH compared to BET while NEVO had an intermediate temperature. All other parameters measured at that time were not affected by diet, environmental temperatures, or their interactions (*p* > 0.05).

The TPA measurements (hardness, springiness, cohesiveness, and chewiness) were higher for the HT group compared to TN (*p* < 0.01, *p* < 0.05, *p* < 0.05, *p* < 0.05, respectively). No differences (*p* > 0.05) in MFI, EJ, and CL were observed among birds due to diets and environmental temperatures. However, a significant increase (*p* < 0.001) occurred in SF in the HT group.

## 4. Discussion

Production parameters declined in the HT group; many studies have reported the negative influence of HT on broilers’ performance [6,7,10,12,39]. In the current study, HS declined DFI and ADG during days 21–35. Similar results were obtained by Bartlett and Smith [40], who reported lower DFI in broilers who experienced cyclic and chronic HS compared to TN birds. The decline in FI could be owing to an attempt by birds to diminish heat created from feed metabolism. Moreover, FCR and PEF were also affected by HS during the finisher phase; this was due to lower body gain and increased mortality rate in the HS group. In the current study, the mortality rate was elevated by HS treatment; the application of HS given rise to 12.0 % mortality compared to 2.5% in the TN treatment. Other investigators supplemented BET at the rate of 0.5 g/kg to diets low in methionine, which is a lower inclusion level than the present study (2 g/kg), and established a significant difference in weight due to BET [41].

While in other studies, BET was statistically similar to the CON diet [42], which agrees with the findings of our present study in that the ADG was not significant from the CON. Only numeric improvements in ADG were reported herein when comparing the CON to BET. It is worth mentioning that we have used the recommended level of methionine in the current study. The other supplement, NEVO, improved ADG significantly over the CON. Comparable results were obtained by Abo El-Fetouh et al. [31]. Moreover, birds received BET or NEVO improved FCR and PEF over the CON. Waldroup et al. [43] reported that with 0.05 or 0.1 % BET supplementation to diets low in methionine, there was improved FCR at 35 and 42 d. This could be due to improved feed utilization in the HS group because of improved osmolality, restored villi length, and water balance in a hypertonic environment. The data strongly suggest that BET works as an osmoregulatory in enteric cells. Klasing et al. [44] presented that the inclusion of BET at 0.05 and 0.1 % improved osmolality and heightened the duodenal villous length. Moreover, BET supplementation at 0.2 % was shown to help the duodenum to sustain water equilibrium in a hypertonic medium [45].

BET supplementation shows some improvement in carcass value. In the current study, BET supplementation improved breast muscle yield over the CON. McDevitt et al. [46] and Waldroup et al.’s [43] findings are also in line with the results of our research. Carcass dressing was improved numerically because of BET and NEVO supplementation. Waldroup and Fritts [47] reported an elevation in dressing percentage in broilers at 42 d supplemented with 0.1 % BET. Moreover, HT decreased breast yield when compared to TN. Similarly, the relative weight of the spleen was affected by temperature; it was lower for the HT group (*p* < 0.01) compared with the TN group. Consistently, the recent work showed that broilers exposed to HS condition experienced a regression in the development of lymphoid organs such as the spleen, bursa, and thymus [48,49]. Besides, the shrinkage in lymphoid organ weights might be attributed to the drop in feed intake, which causes fewer nutrients for the proper growth of these organs under HS conditions [40].

Meat pH is an essential factor in meat quality measurements that happens during the stiffness of muscles; it can influence the texture, color, and WHC of meat. Within the first 15 min post-mortem, pH is a vital constituent to define the shelf life of meat products [50]. Usually, a fast drop in meat pH can bring on denaturation of proteins, which might give rise to pale color and low-WHC of meat. Various studies suggested that nanoemulsion-based products can affirmatively influence breast muscles’ physicochemical and sensory attributes [18]. Unfortunately, there are very few reports in the literature that examined NEVO’s effect on meat attributes. In the present research, BET had a more substantial impact on fillets’ physicochemical characteristics compared to NEVO. Kpodo et al.’s [51] report showed that BET had no effect on breast meat color, but HS elevated lightness and reduced yellowness of breast meat. However, Akşit et al. [52] showed that when broilers were treated with cyclic HS, the lightness of their pectoral meat was elevated but not the yellowness. The MFI symbolizes destruction to myofibrillar proteins induced by homogenization, and it is associated with other indices for muscles, such as SF, which was higher for the HT group [53]. TPA measurements represent sensory attributes of meat that imitate the circumstances to which meat is undergone within a mouth. All parameters were worsening by the HT factor.

## 5. Conclusions

Collectively, the results of this study support that HT at 35°C has a negative impact on feed intake, body growth, and conversion ratio when applied during the finisher period. Moreover, HT decreased breast yield, increased fillets temperature, and lowered fillets pH_15min_ compared to the TN group. BET or NEVO supplementation during days 21–35 old improved FCR and PEF over the CON. BET improved breast fillets temperature pH_15min_, and pH_24hr_ compared to the CON, while NEVO had an intermediate temperature. Higher pH values for breast fillets were obtained from the BET group. This indicates that supplementing water with BET improved performance and meat characteristics while NEVO supplementation improved growth performance.

## Figures and Tables

**Table 1:** Ingredients (on fed basis) and calculated nutrients of broilers basal finisher diet.

Ingredient	Finisher (%)	Analysis	(%)
Yellow corn	58.09	ME, kcal/kg	3200
Soybean meal	32.15	Crude protein, %	20
Wheat bran	2.20	Non-phytate P, %	0.36
Corn oil	4.20	Calcium, %	0.81
Dicalcium Phosphate DCP	1.62	Digestible Lysine, %	1.03
Ground Limestone	0.79	Digestible Sulfur amino acids, %	0.80
Salt	0.40	Digestible Threonine, %	0.69
DL-methionine	0.25		
Lysine-HCL	0.11		
Vitamin- Mineral premix ^1^	0.20		
Total	100		

^1^ Provides per kg diet: Vitamin A, 2, 400,000 IU; vitamin D, 1, 000,000 IU; vitamin E, 16,000 IU; vitamin K, 800 mg; vitamin B1, 600 mg; vitamin B2, 1600 mg; vitamin B3, 8000 mg; vitamin B5, 3000 mg; vitamin B6, 1000 mg; vitamin B7, 40 mg; vitamin B9, 400 mg; vitamin B12, 6 mg; antioxidants, 3000 mg; Co, 80 mg; Cu, 2000 mg; I, 400; Fe, 1200 mg; Mn, 18000 mg; Se, 60 mg, and Zn, 14,000 mg.

**Table 2:** Effects of the treatments on performance parameters of broiler chickens during finisher phase.

Parameters ^1^		Treatments ^2^						SEM ^3^	*p*-Value ^4^		
		TN			HT						
		CON	NEVO	BET	CON	NEVO	BET		TEMP	DIET	TEMP × DIET
Days 21–28	DFI (g)	137	137	135	135	134	130	1.80	NS	NS	NS
	ADG (g)	94.6	101.4	99.5	93.7	96.4	93.7	1.88	0.050	NS	NS
	FCR (g: g)	1.45	1.35	1.36	1.44	1.39	1.39	0.018	NS	0.001	NS
	PEF	350	388	382	337	370	365	7.40	0.050	0.001	NS
Days 28–35	DFI (g)	144	142	141	137	141	137	1.90	NS	NS	NS
	ADG (g)	86.8	92.4	90.4	82.1	88.0	84.6	1.61	0.010	0.050	NS
	FCR (g: g)	1.66	1.54	1.56	1.69	1.60	1.61	0.017	0.010	0.001	NS
	PEF	345	377	368	320	358	342	6.97	0.010	0.001	NS
Days 21–35	DFI (g)	141	139	138	137	137	134	1.64	0.050	NS	NS
	ADG (g)	90.4	96.6	94.6	87.4	91.9	88.8	1.43	0.010	0.050	NS
	FCR (g: g)	1.56	1.45	1.46	1.57	1.50	1.51	0.013	0.050	0.001	NS
	PEF	367	401	393	356	382	373	6.41	0.050	0.010	NS
Day 35	BW (kg)	2.00	2.03	2.00	1.95	2.00	1.96	0.030	NS	NS	NS

^1^ DFI, daily feed intake; ADG, average daily gain; FCR, feed conversion ratio; PEF, performance efficiency factor; BW, body weight. ^2^ TN, thermoneutral environment (24 ± 1 °C); HT, high-temperature environment (35 ± 1 °C); CON, control; NEVO, nano-emulsified vegetable oil (5 mL/L drinking water); BET, betaine (2 g/L drinking water). ^3^ SEM, pooled standard error of the means. ^4^ TEMP, temperature effect; NS, not significant.

**Table 3:** Effects of the treatments on the relative weight (%) of carcass yield and internal organs at 35 d.

Parameters	Treatments ^2^						SEM ^3^	*p*-Value ^4^		
	TN			HS						
	CON	NEVO	BET	CON	NEVO	BET		TEMP	DIET	TEMP × DIET
Dressing	70.6	71.8	71.7	70.3	70.6	71.0	0.590	NS	NS	NS
Breast	30.8	32.3	32.6	30.5	30.3	31.8	0.520	0.050	0.050	NS
Leg	21.1	21.2	20.4	20.9	20.9	20.6	0.330	NS	NS	NS
Fat	0.730	0.758	0.702	0.702	0.926	0.840	0.089	NS	NS	NS
Liver	2.40	2.50	2.30	2.40	2.40	2.00	0.090	NS	0.050	NS
Gizzard	2.20	2.20	2.00	2.40	2.30	2.20	0.150	NS	NS	NS
IRW ^1^	2.79	2.80	2.63	2.56	3.08	2.40	0.140	NS	0.050	NS
Bursa	0.132	0.258	0.242	0.144	0.158	0.178	0.023	0.050	0.010	NS
Spleen	0.106	0.102	0.124	0.088	0.070	0.092	0.010	0.010	NS	NS
Thymus	0.220	0.230	0.288	0.218	0.236	0.274	0.009	NS	NS	NS

^1^ IRW, intestine relative weight. ^2^ TN, thermoneutral environment (24 ± 1 °C); HT, high-temperature environment (35 ± 1 °C); CON, control; NEVO, nano-emulsified vegetable oil (5 mL/L drinking water); BET, betaine (2 g/L drinking water). ^3^ SEM, pooled standard error of the means (*n* = 10). ^4^ TEMP, temperature effect; NS, not significant.

**Table 4:** Effect of the treatments on meat quality of pectoralis major muscles of broiler chickens sampled at 35 d.

Parameters ^1^			Treatments ^2^						SEM ^3^	*p*-Value ^4^		
			TN			HS						
			CON	NEVO	BET	CON	NEVO	BET		TEMP	DIET	TEMP × DIET
15 min post-mortem	pH		6.20	6.22	6.32	6.12	6.12	6.20	0.042	0.010	0.050	NS
	Temperature		31.1	30.7	30.8	33.9	33.1	32.0	0.340	0.001	0.050	NS
	Color items	L*	48.3	47.7	49.2	49.3	47.3	49.4	1.56	NS	NS	NS
		a*	3.75	2.72	2.37	2.34	2.45	2.08	0.420	NS	NS	NS
		b*	5.09	3.52	3.85	5.03	3.94	4.51	0.680	NS	NS	NS
		C*	6.38	4.51	4.70	5.59	4.74	5.05	0.630	NS	NS	NS
		H°	53.0	49.5	55.5	63.8	56.6	66.8	5.85	NS	NS	NS
		a*: b*	0.788	0.918	0.848	0.508	0.710	0.450	0.193	NS	NS	NS
24 h post-mortem	pH		5.98	6.02	6.10	5.90	6.02	6.08	0.043	NS	0.010	NS
	Color items	L*	51.7	52.1	53.1	53.7	51.9	52.6	1.19	NS	NS	NS
		a*	4.31	3.87	4.78	3.64	3.75	3.66	0.545	NS	NS	NS
		b*	7.77	8.66	7.99	9.01	7.71	8.90	0.784	NS	NS	NS
		C*	9.01	9.55	9.38	9.74	8.64	9.69	0.769	NS	NS	NS
		H°	61.0	66.5	57.8	68.0	63.8	66.8	3.61	NS	NS	NS
		a*: b*	0.578	0.442	0.644	0.406	0.502	0.442	0.084	NS	NS	NS
		∆E*	2.41	3.23	3.09	3.05	3.03	2.96	0.340	NS	NS	NS
MFI			79.2	82.6	82.4	73.0	81.6	86.7	3.40	NS	NS	NS
EJ			0.282	0.272	0.278	0.312	0.306	0.288	0.017	NS	NS	NS
CL			30.1	32.6	30.1	35.1	32.0	29.0	1.71	NS	NS	NS
SF			1.09	1.01	0.97	1.55	1.71	2.24	0.192	0.001	NS	NS
TPA		hardness	0.644	0.772	0.722	1.08	0.900	1.00	0.123	0.010	NS	NS
		springiness	0.834	0.854	0.834	0.990	0.898	0.900	0.043	0.050	NS	NS
		cohesiveness	0.484	0.478	0.496	0.540	0.510	0.508	0.015	0.050	NS	NS
		chewiness	0.266	0.336	0.310	0.590	0.430	0.510	0.070	0.050	NS	NS

^1^ L*, lightness; a*, redness; b*, yellowness; C*, chroma; H°, hue angle; ∆E*, color difference; MFI, myofibril fragmentation index; EJ, expressed juice; CL, cooking loss; SF, shear force; TPA, texture profile analysis. ^2^ TN, thermoneutral environment (24 ± 1 °C); HT, high-temperature environment (35 ± 1 °C); CON, control; NEVO, nano-emulsified vegetable oil (5 mL/L drinking water); BET, betaine (2 g/L drinking water). ^3^ SEM, pooled standard error of the means (*n* = 10). ^4^ TEMP, temperature effect; NS, not significant.
